# Hepatitis A Virus Genotype Distribution during a Decade of Universal Vaccination of Preadolescents

**DOI:** 10.3390/ijms16046842

**Published:** 2015-03-25

**Authors:** Lucía D’Andrea, Francisco J. Pérez-Rodríguez, Montserrat de Castellarnau, Sandra Manzanares, Josep Lite, Susana Guix, Albert Bosch, Rosa M. Pintó

**Affiliations:** 1 Enteric Virus Laboratory, Department of Microbiology and Institute of Nutrition and Food Safety, University of Barcelona, Diagonal 643, 08028 Barcelona, Spain; E-Mails: luciadandrea@ub.edu (L.D.); fjperez@ub.edu (F.J.P.-R.); mdecastellarnau@ub.edu (M.C.); susanaguix@ub.edu (S.G.); abosch@ub.edu (A.B.); 2 Public Health Agency of Barcelona, Plaça Lesseps 1, 08023 Barcelona, Spain; E-Mail: smanzana@aspb.cat; 3 Microbiology Unit, CatLab, 08232 Viladecavalls, Spain; E-Mail: jlite@catlab.cat

**Keywords:** hepatitis A, HAV genotypes, age-group, children, MSM, vaccine-escape variants, vaccination

## Abstract

A universal vaccination program among preadolescents was implemented in Catalonia, Spain, during the period of 1999–2013 and its effectiveness has been clearly demonstrated by an overall significant attack rate reduction. However, reductions were not constant over time, and increases were again observed in 2002–2009 due to the occurrence of huge outbreaks. In the following years, in the absence of large outbreaks, the attack rate decreased again to very low levels. However, an increase of symptomatic cases in the <5 age group has recently been observed. This is an unexpected observation since children younger than 6 are mostly asymptomatic. Such a long vaccination campaign offers the opportunity to analyze not only the effectiveness of vaccination, but also the influence of the circulating genotypes on the incidence of hepatitis A among the different age groups. This study has revealed the emergence of genotype IC during a foodborne outbreak, the short-lived circulation of vaccine-escape variants isolated during an outbreak among the men-having-sex-with-men group, and the association of genotype IIIA with the increase of symptomatic cases among the very young. From a public health perspective, two conclusions may be drawn: vaccination is better at an early age, and the vaccination schedule must be complete and include all recommended vaccine doses.

## 1. Introduction

In low-moderate endemic countries, introduction of hepatitis A virus (HAV) occurs mainly through immigration flows, traveling to endemic areas or consuming imported foods from such endemic areas [1,2,3]. Additionally, once introduced in a given area, circulating strains are transmitted through person-to-person contact, and among these the men-having-sex-with-men (MSM) group is a relevant target due to risky practices of fecal-oral transmission [4,5,6].

Hepatitis A vaccination is a good measure to prevent the re-emergence of the infection in non-endemic countries [7]. Available vaccines cover all genotypes so far described since only a single serotype of HAV exists in spite of its genomic diversity [7]. A vaccination program among preadolescents was implemented in Catalonia from 1999 to 2013, and its effectiveness was clearly demonstrated by an attack rate reduction from 6.2 cases per 100,000 inhabitants in 1996–1998 to 2.6 in 1999–2001 [8]. However, increases in the attack rate up to values over 3 were again observed in 2002–2009 [1,8]. These increases were associated with several outbreaks: two in the MSM group in 2003–2004 [5] and in 2009 [9], respectively, and a third shellfish-borne outbreak in 2008 [10]. In the following years, in the absence of large outbreaks, the attack rate decreased again to very low levels (1.68 on average). However, an increase of symptomatic cases in the <5 age-group has lately been observed. This is an unexpected observation since children younger than 6 are mostly asymptomatic, with only 10% of them showing jaundice, as reported by the World Health Organization (http://www.who.int/mediacentre/factsheets/fs328/en/). In contrast, hepatitis A infection severity increases with the patient’s age.

The influence of the vaccination campaign in the pre-adolescence group in Catalonia on the attack rate and the evolution of the genotype distribution during the 2004–2013 period will be analyzed in the present work. The emergence of genotypes previously uncommon in the region and the circulation of low-fitness vaccine-escape variants will be discussed and relevant conclusions for the implementation of public health measures will be drawn.

## 2. Results and Discussion

### 2.1. Effectiveness of the Universal Vaccination Campaign Revealed through the Evolution of the Global and Age-Group Specific Attack Rates

The vaccination campaign started in 1999 targeting preadolescents at the age of 12; consequently in 2004, 2005, 2006, 2007, 2008, 2009, 2010, 2011, 2012 and 2013 the protected population covered those 12–17, 12–18, 12–19, 12–20, 12–21, 12–22, 12–23, 12–24, 12–25 and 12–26 years old, respectively.

**Figure 1:**
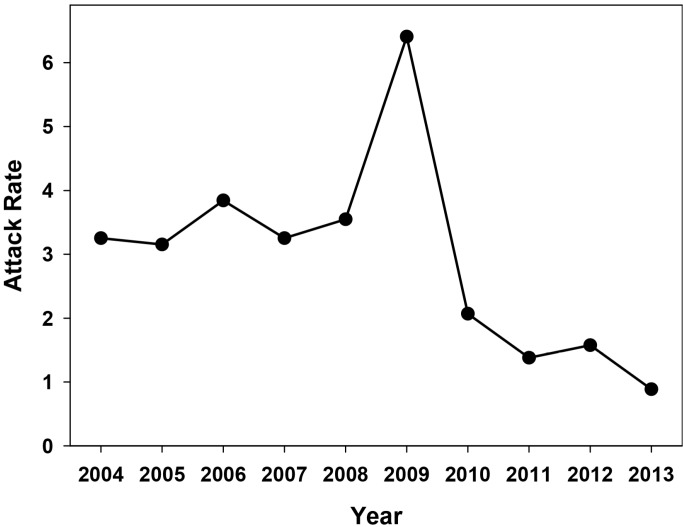
Hepatitis A attack rates in the decade 2004–2013 in Catalonia, Spain, in the context of a universal vaccination campaign for preadolescents at the age of 12 years. Data is distributed in natural years. Attack rates are defined as the number of cases per 100,000 inhabitants.

**Figure 2:**
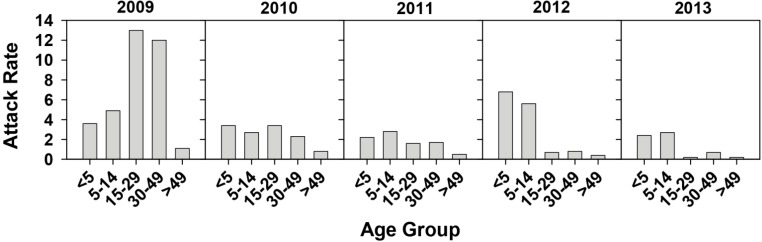
Hepatitis A attack rates for different age groups during the 2009–2013 period in Catalonia, Spain, in the context of a universal vaccination campaign for preadolescents at the age of 12 years. Data is distributed in natural years. Attack rates are defined as the number of cases per 100,000 inhabitants of each age group.

An analysis of the global annual attack rate (number of hepatitis A cases per 100,000 inhabitants) in 2004–2013 in Catalonia was performed to ascertain the long-term effectiveness of the vaccination campaign, which lasted fifteen years (Figure 1). The effectiveness of the vaccination campaign was generally good, with an average attack rate of 2.98 ± 1.61 during this decade. However, two different patterns were observed (Figure 1). From 2004 to 2008, the average attack rate was 3.5 ± 0.32, while in 2010–2013 it was significantly lower (*p* < 0.05) at 1.5 ± 0.05. These two periods were separated by an extremely high attack rate of 6.5 in 2009 which was caused by a huge outbreak among the MSM group [9]. During this outbreak, vaccine-escape virus variants were isolated [11], which could negatively impact the future efficacy of the vaccine. To evaluate this possibility, an analysis of the attack rate distribution in the different age groups during the 2009–2013 period was undertaken to look for a rise in the incidence of hepatitis A specifically in groups with a vaccinated population (Figure 2).

**Figure 3:**
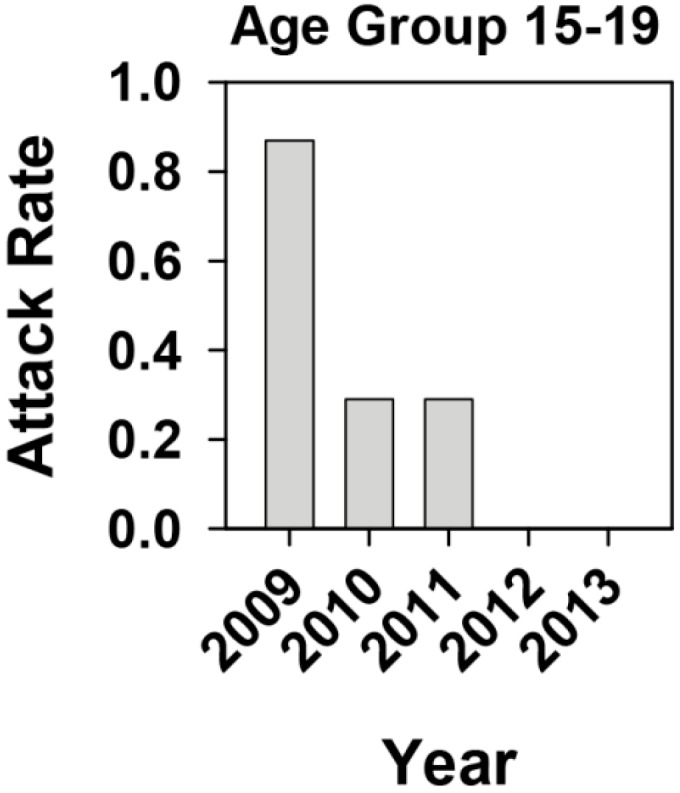
Hepatitis A attack rate of the 15–19 age group during the 2009–2013 period in Catalonia, Spain, in the context of a universal vaccination campaign for preadolescents at the age of 12 years. The 15–19 age group mostly includes young adults who have been vaccinated. Attack rates are defined as the number of cases per 100,000 inhabitants of the 15–19 age group.

This analysis revealed, as expected, a high attack rate in the 15–29 and 30–49 age groups in 2009, due to the outbreak. Since the 15–29 age group was not totally vaccinated, a deeper analysis of the 15–19 teenage group, whose members were all theoretically protected by the vaccine, was performed. This analysis revealed attack rates of 0.87 (3 cases), 0.29 (1 case), 0.29 (1 case), 0 and 0 per 100,000 inhabitants of this age in 2009, 2010, 2011, 2012 and 2013, respectively (Figure 3).

Only one (a 19-year-old man) out of three detected cases in 2009 in the 15–19 years old vaccinated group was directly involved in the MSM outbreak. The remaining two cases in 2009 (a man and a woman) and those cases in 2010–2011 (a man and a woman) were not in the MSM group. Similarly, vaccine-escape variants were also isolated from non-vaccinated patients outside the MSM group, in 2009 but not thereafter. Altogether, these results indicate that vaccine-escape variants isolated in 2009, in the context of the MSM outbreak, were transferred into the general population, circulated for a period of time and finally were outcompeted by vaccine-sensitive strains, likely due to their very low fitness [11]. In fact, the vaccine-escape variants were first isolated from HIV-positive patients vaccinated against HAV but who had not received all required vaccine doses, the ideal situation for the isolation of such variants with higher and lower fitness than the sensitive-type strains in the presence and absence of antibodies, respectively [12]. Additionally, the reported case in 2011 seemed to be a vaccine failure rather than a case linked to a vaccine variant. The patient was a 15-year-old girl who received the complete vaccine schedule three years before the onset of the disease (according to their personal vaccine registry), and the isolated strain did not harbor mutations at the epitope regions of the immunodominant site or the glycophorine A binding sites [13,14] which were analyzed after sequencing fragments of the VP3 and VP1 coding regions. Similarly, a second vaccine failure was reported in 2012 for a 25-years old female who also received the complete vaccine schedule 13 years before the onset of the disease (according to their personal vaccine registry), and again the isolated strain did not show mutations at the antigenic sites. Since during this decade almost 1 million two-dose vaccines were administered to preadolescents at the age of 12 and only two confirmed vaccine failures have been reported among healthy patients, a failure attack rate of 2 per million vaccinated individuals may be estimated. Vaccine failures have been described in other occasions [15]. The very low attack rate seen in the period 2010–2013 (Figure 1) was likely due to the effectiveness of the universal preadolescent vaccination campaign and to a certain extent to intervention programs specifically directed at the MSM group. Such interventions included voluntary vaccination of those older than 23, 24, 25 and 26 years in 2010, 2011, 2012 and 2013, respectively, which were not covered by the universal program, and the implementation of intensive informative campaigns (http://www.aspb.cat/quefem/documents_sida_hepatitis.htm; [5]).

**Figure 4:**
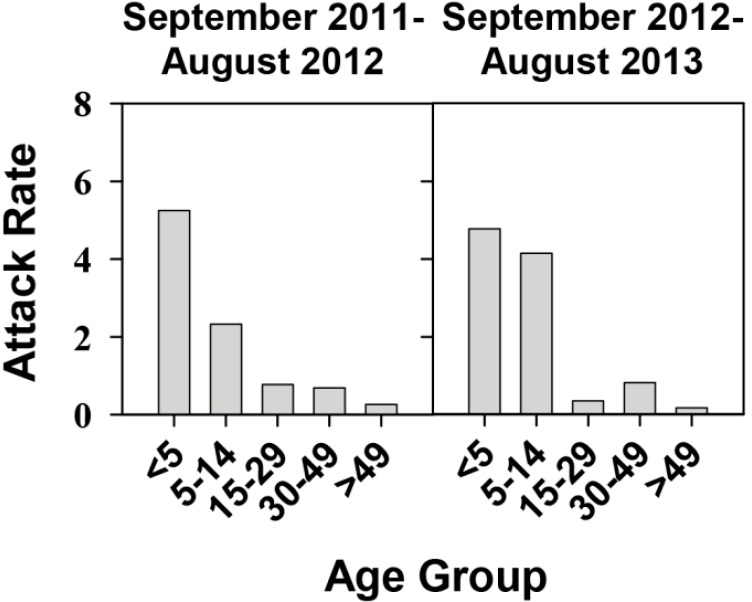
Hepatitis A attack rates of different age groups during September 2011–August 2013 in Catalonia, Spain, in the context of a universal vaccination campaign of preadolescents at the age of 12 years. Data is distributed in two sets: September 2011–August 2012 and September 2012–August 2013. Attack rates are defined as the number of cases per 100,000 inhabitants of each age group.

The attack rate distribution in the different age groups revealed an additional point: an abrupt increase of symptomatic cases among the 0–5 age group during the year 2012 (Figure 2). This rise of symptomatic cases induced an increase of the attack rate up to 6.8 per 100,000 inhabitants of this age-group compared to other years with attack rates in the range 2.2–3.6 (Figure 2). During 2012, the attack rate in the 5–14 group was also quite high at 5.6 (Figure 2). However, looking more precisely at the period September 2011–August 2012, the attack rate of the <5-year group was 5.25, while the attack rate of the 5–14 group was lower, at 2.33 (Figure 4).

During this period, a total of 73 hepatitis A cases were declared and distributed as follows: 22 cases in the <5 group, 18 in the 5–14 group, 9 in the 15–29 group, 17 in the 30–49 group, and 7 in the >49 age groups, respectively. This distribution shows that 30%, 25%, 12%, 23% and 10% of all declared hepatitis A cases were in the <5, 5–14, 15–29, 30–49 and >49 age-groups, respectively, while, in contrast, the population in these groups represents, 6%, 10%, 16%, 33% and 35% of total population in Catalonia in this period. The expected proportions of hepatitis A cases would be much lower for the <5 years group and much higher for the 30–49 age group, since in the former group most cases should be asymptomatic, while in the latter, most of the individuals are non-vaccinated and expected to be immunologically naïve for their age.

During the following year-period (September 2012–August 2013), the attack rate in the <5 group remained high at 4.77, with 20 cases, while the attack rate in the 5–14 group also increased to 4.14 (Figure 4), with 32 cases. In fact, the average attack rate of the group <5 years during the period September 2011-August 2013 (5.0 ± 0.3) was significantly (*p* < 0.05) higher than the average of the same group during the years 2009–2010 (3.5 ± 0.1).

### 2.2. Genotype Analysis

**Figure 5:**
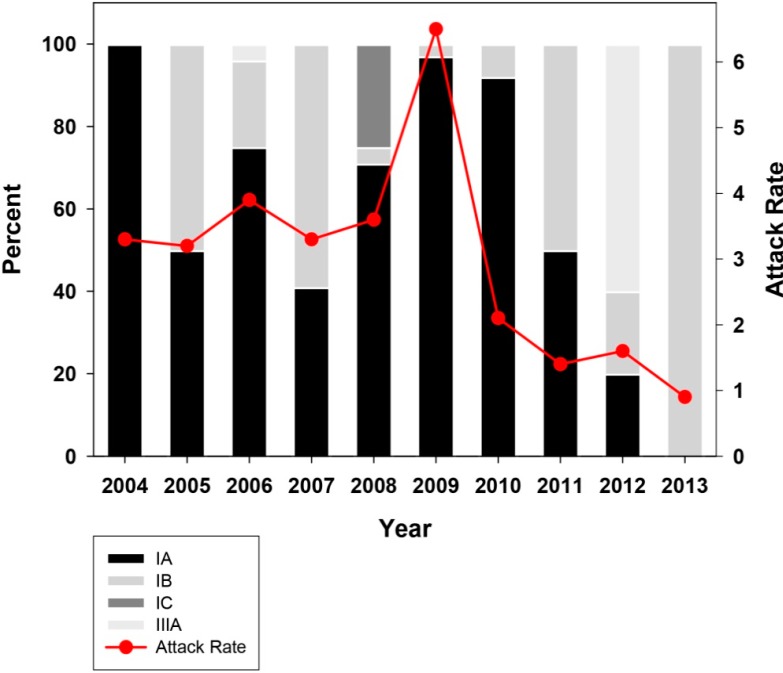
Distribution of HAV genotypes in Catalonia, Spain, in the decade 2004–2013. A universal vaccination campaign of preadolescents at the age of 12 years started in 1999 and finished in 2013. Data is distributed in natural years. Genotypes are molecularly defined using the VP1X2A genomic region. Attack rates are defined as the number of cases per 100,000 inhabitants.

An analysis of the distribution of the different circulating genotypes during the decade under study was performed using the sequence from the VP1/2A junction region from 160 isolates, out of 215 randomly selected serum samples tested, which represented 9.5% of all declared cases. Strains belonging to genotype I (IA, IB and IC) and III (IIIA but not IIIB) were detected. Genotype II strains did not circulate in Catalonia; in fact, it is an uncommon genotype outside the sub-Saharan continent although it has sporadically being detected in France [16]. The percent of circulating IA, IB and IC subgenotypes changed over time (Figure 5). Overall, the most common subgenotype during this decade in Catalonia was IA (66.6% ± 33.3%), as is the case in the United States and Western Europe [17,18], followed by subgenotype IB (24.5% ± 34.2%) which is very common in North Africa and the Middle-East [1,2]. Subgenotype IC was detected only in 2008 but was quite abundant that year (25%) due to the occurrence of a shellfish-borne outbreak [1,10], and subgenotype IIIA was detected associated to a sporadic case in 2006 (4%) and in 2012 as the most frequent type (60%).

**Figure 6:**
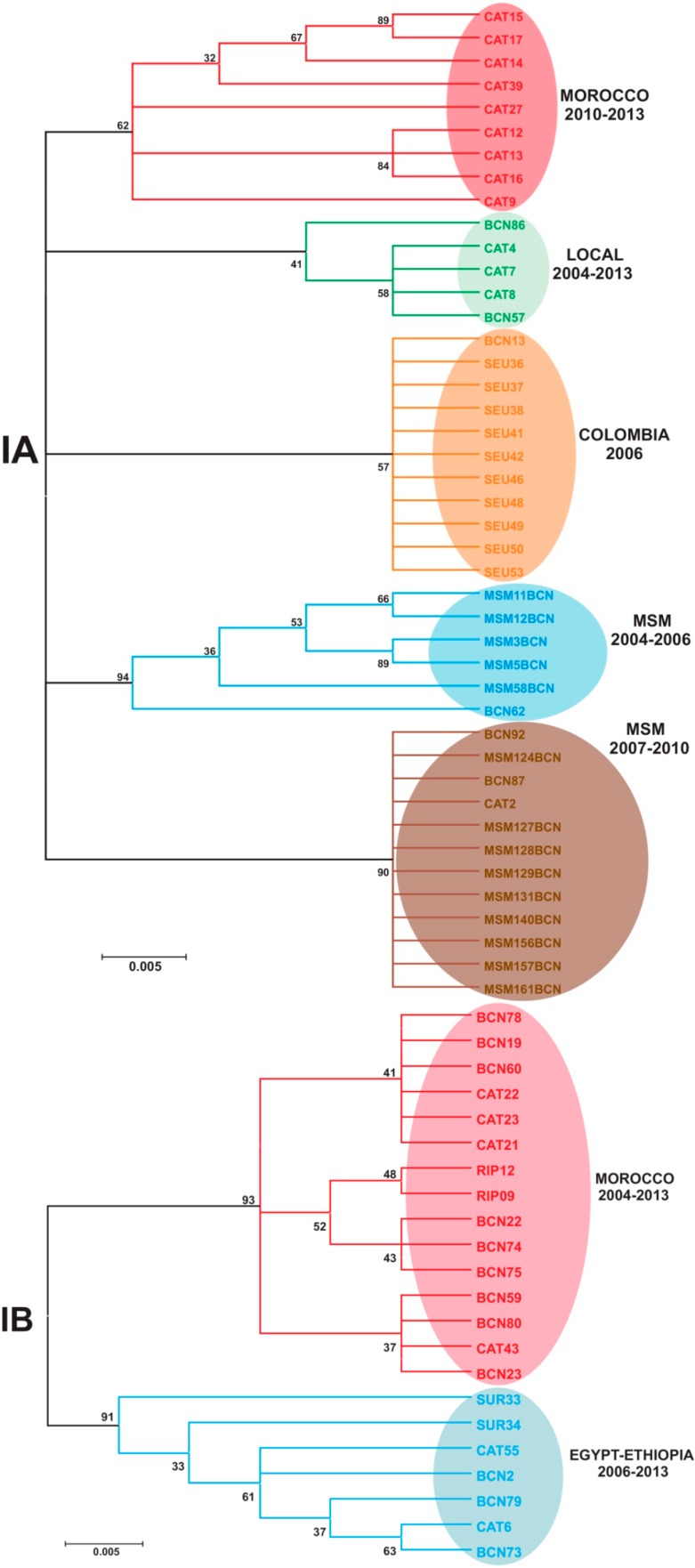
Phylogenetic trees based on the VP1/2A junction region, from strains belonging to subgenotype IA and IB. Neighbor-Joining, Kimura 2-parameter, bootstrap 1000 replicates.

**Figure 7:**
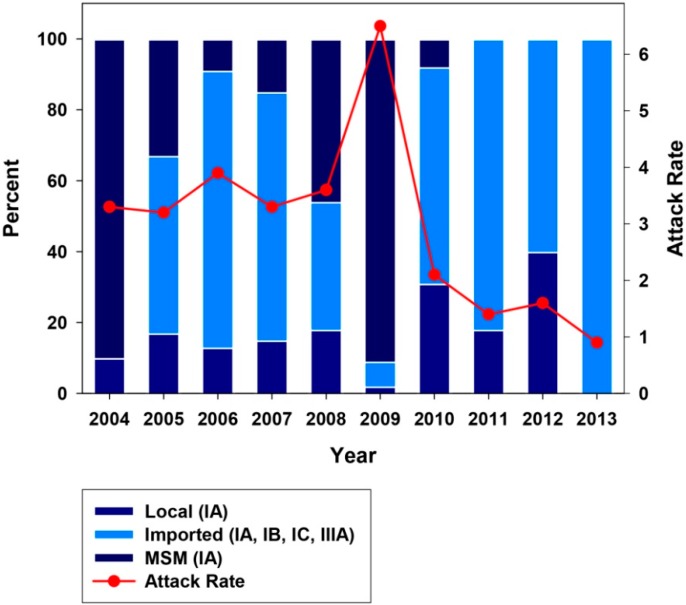
Origin of the HAV strains isolated in the decade 2004–2013. Data is distributed in natural years. Attack rates are defined as the number of cases per 100,000 inhabitants.

A phylogenetic analysis of the multiple strains belonging to subgenotype IA and IB was undertaken to track their geographical origin (Figure 6). Despite the vaccination campaign, few local strains all belonging to subgenotype IA were still circulating in Catalonia during this decade (Figure 6 and Figure 7). The very high proportions of subgenotype IA in years 2004, 2008 and 2009 were due to the introduction of endemic strains of the MSM group [4,5], while in 2006 and 2010 they were mainly due to the introduction of strains from Colombia and Morocco, respectively (Figure 6 and Figure 7). Subgenotype IB strains were common in years 2005, 2006, 2007, 2011 and 2013, introduced from Morocco, Egypt and Ethiopia (Figure 6 and Figure 7). While IB strains imported from Morocco are common in our area, the importation of IA strains is something new and seen only since 2010, but previously reported in other countries [2,19]. The relevant high proportion of subgenotype IIIA strains (60% of all typed strains) in 2012 corresponded to the above mentioned significant rise of symptomatic cases among the very young. In fact, all cases were in children, with an average age of 3.6 ± 1.7 years. Genotype IIIA is uncommon in Europe and the US [2,3,18], although a certain increase has lately been detected in the European Nordic countries [20], as well as in Japan [21]. In contrast, genotype IIIA is very common in the Asian continent and particularly in Pakistan [22]. In fact, all IIIA strains isolated in 2012 in Catalonia were from children returning from Pakistan. Based on a 324-nt region from the VP1 region (nt 2475-2798 of the HM175 strain) the closest reference IIIA strains to the ones hereby isolated (GIIIABCN; Accession number KJ412329) were the AB279732 from Japan and the AJ299464 from Norway [23]. They were also closely related to strain FJ360735 isolated in India in 1997 [24]. In contrast, they were more distantly related to the HQ401239 strain from Catalonia that was isolated in 2006 from an 11-year old Roman boy [1]. Genotype IIIA’s clinical severity is controversial. Some studies suggest a more severe outcome in adult patients [25] compared to genotype IA, while others report no differences [26]. In our hands, this genotype IIIA strain seems to be associated with an increase of symptomatic cases among the very young, although no clinical signs of increased severity were reported. No such relationship has been described before [2,20], so it is unknown if this is a common trait of all genotype IIIA strains or specifically relates to this strain. However, in India, where genotype IIIA is the most prevalent, 52% of the isolates from clinical cases are from the <5 group, 31% from the 6–14 age group and 12% from the adult group [27], suggesting that a tendency to produce clinical symptoms in the very young children may be common to all genotype IIIA strains. These findings may have important implications for the age of vaccination. While in the US, hepatitis A vaccination is recommended to be integrated into the routine childhood vaccination schedule at the age of 12–23 months (http://www.cdc.gov/vaccines/pubs/pinkbook/hepa.html; [28]), in Catalonia (Spain) [8] or Puglia (Italy) [29] vaccination programs have been implemented at schools for 12 year-olds. Data included herein indicate that an earlier administration of the vaccine would be highly beneficial to protect the youngest group, who may be more susceptible to developing clinical symptoms than previously anticipated, at least from imported genotype IIIA strains.

Despite these particular occurrences, the overall effectiveness of the vaccination program in preventing symptomatic hepatitis A infections in Catalonia was good, and correlated with the reported high anti-HAV prevalence in the vaccinated adolescent population [30]. Actually, good seroconversion is the rule in most vaccination programs for adolescents and toddlers in other countries [31]. Thus, it can be concluded that vaccination is the best option to prevent the re-emergence of hepatitis A cases in developed countries, but it should be emphasized that the complete recommended schedule of vaccination must be carried out and that the earlier the administration of the vaccine, the better the population protection.

## 3. Experimental Section

### 3.1. Estimation of the Attack Rates

For the estimation of the hepatitis A attack rates, defined as the number of clinical cases of hepatitis A per 100,000 total inhabitants or the number of clinical cases in a specific age group per 100,000 inhabitants of that group, public databases from the Generalitat de Catalunya were used. One is the epidemiological surveillance bulletin of Catalonia (BEC; http://www20.gencat.cat/portal/site/canalsalut/menuitem.21c58aea29b124fc48af8968b0c0e1a0/?vgnextoid=31dadc4beb235310VgnVCM2000009b0c1e0aRCRD&vgnextchannel=31dadc4beb235310VgnVCM2000009b0c1e0aRCRD) which provides information on the number of hepatitis A cases per week, gender and age-groups (<5, 5–9, 10–14 15–19, 20–29, 30–39, 40–49, 50–59 and >60), and another is the demography network of Catalonia (idescat; http://www.idescat.cat/) which provides detailed information of the number of inhabitants per year, gender and age. For the sake of clarity in most of our analysis, the number of age groups was reduced to five (<5, 5–14, 15–29, 30–49 and >49), with the exception of the analysis of the attack rate of the 15–19 age group which was, theoretically, vaccinated.

Statistical differences between the attack rates of the periods 2004–2008 and 2010–2013 and between the attack rates in the <5 age group during 2009–2010, September 2011–August 2012 and September 2012–August 2013 were assessed using the Student *t*-test (unpaired).

### 3.2. Epidemiological Data and Genotype Determination

Two hundred and fifteen (*n* = 215) serum specimens were collected from anti-HAV IgM-positive patients in Catalonia during the decade 2004–2013, including sporadic and outbreak cases, which represented 12.5% of total declared cases. Epidemiological data including age, sex, vaccination status, suspected source of infection, recent travels, mode of transmission (person to person, sexual contact, intravenous drug administration) and sexual orientation were recorded for all patients. The University of Barcelona (Institutional Review Board IRB 00003099) Bioethics Committee approved the study and waived the need for consent. All serum samples were collected as part of standard patient care. Samples used were anonymously provided and identified through restricted codes. All data were treated in a strictly confidential manner following the ethical principles of the Helsinki Declaration of 1964 revised by the World Medical Organization in Tokyo, 2008 and the Organic Law 15/1999 of Data Protection in Spain.

For the characterization of the epitope regions of the immunodominant and the glycophorine A binding sites of vaccine-escape mutants and strains from vaccine failures, the complete sequence for the VP1 and VP3 coding regions was obtained, while the VP1/2A junction was sequenced from all isolates and used for the phylogenetic genotype determination [32]. RNA was extracted from 150 μL of serum samples with the Nucleospin RNA virus kit (Macherey Nagel, Düren, Germany). HAV RNA was amplified by RT-PCR using the Expand Reverse Transcriptase and the Expand HiFi PCR System enzyme kits (Roche, Barcelona, Spain) and previously described primers [13,14,33,34]. Nucleotide sequences were compared with those deposited in Genbank and EMBL using the BLAST N (Bethesda, MD, USA) and CLUSTAL W (Cambridge, UK) softwares. Boot-Strap phylogenetic trees were constructed using the MEGA (Tempe, AZ, USA) software package, version 4.0.2.

### 3.3. Accession Numbers

Sequences have been deposited in GenBank with accession numbers HQ401214 to HQ401267, KJ412329 and KP702119 to KP702137.

## 4. Conclusions

Hepatitis A has drastically declined in Catalonia since 1999 thanks to the implementation of a universal vaccination program among preadolescents at the age of 12, with average attack rates as low as 1.5 per 100,000 inhabitants in the 2010–2013 period.

Vaccine failures occur at a very low rate. However, when vaccination schedules are not complete, particularly in immunocompromised patients, the chances of isolating vaccine-escape variants increase, such the ones herein described in the context of a huge outbreak among the MSM group in 2009. Fortunately, these escape variants were further outcompeted by vaccine-sensitive types and are no longer being detected.

Although young children under 6 years of age usually show asymptomatic hepatitis A infections, genotype IIIA strains may produce more clinical cases in this age group than previously anticipated. Consequently, vaccination of toddlers is recommended to prevent clinical cases in very young children.

Molecular typing tools combined with classical epidemiological data are required for a comprehensive analysis of the efficacy of vaccination campaigns.
